# Host plant affects the sexual attractiveness of the female white-spotted longicorn beetle, *Anoplophora malasiaca*

**DOI:** 10.1038/srep29526

**Published:** 2016-07-14

**Authors:** Hiroe Yasui, Nao Fujiwara-Tsujii

**Affiliations:** 1 Laboratory of Insect Behaviour, National Institute of Agrobiological Sciences (NIAS), Ohwashi 1-2, Tsukuba, Ibaraki 305-0851, Japan

## Abstract

*Anoplophora malasiaca* (Coleoptera: Cerambycidae) is a serious pest that destroys various landscape and crop trees in Japan. We evaluated the precopulatory responses of three different *A. malasiaca* populations collected from mandarin orange, willow and blueberry trees. Most of the males accepted mates from within the same host plant population as well as females from the willow and blueberry populations. However, significant number of males from the blueberry and willow populations rejected females from the mandarin orange population immediately after touching them with their antennae. Because all three of the female populations produced contact sex pheromones on their elytra, the females of the mandarin orange population were predicted to possess extra chemicals that repelled the males of the other two populations. *β*-Elemene was identified as a key component that was only found in mandarin orange-fed females and induced a rejection response in willow-fed males. Our results represent the first example of a female-acquired repellent against conspecific males of different host plant populations, indicating that the host plant greatly affects the female’s sexual attractiveness.

Infestations of two devastating insect pests, the Asian longhorned beetle, *Anoplophora glabripennis* (Motschulsky), and the citrus longhorned beetle, *Anoplophora chinensis* (Forster) (Coleoptera: Cerambycidae), which are native to parts of Asia, have been reported in North America and Europe. These two beetles are now serious threats to landscape trees because they often kill their host trees owing to their vigorous feeding during their larval stages^1^. Moreover, the Asian longhorned beetle is included in a list of the 100 worst invasive species^2^. In regions where the beetle has been introduced, aggressive containment programs have been initiated, such as the removal and destruction of any tree having signs of beetle infestation^3^.

The white-spotted longicorn beetle, *Anoplophora malasiaca* (Thomson), is widely distributed in Japan^4^,^5^. *A. malasiaca* is a serious pest of horticultural crops, such as citrus, apple, and pear, as well as street trees, such as the oriental plane tree and willow^4^,^6^. This species has a very wide host plant range, which includes 108 known species of trees belonging to 73 genera^7^. The control of this species is highly desirable in Japan, as is the case with *A. glabripennis* and *A. chinensis* in North America and Europe^1^,^3^.

Recently, extensive progress has been made in understanding the *A. malasiaca* mate location and recognition system in adults collected from mandarin orange [MO; *Citrus unshiu* Marc. (Rutaceae)], blueberry (BB; *Vaccinium* spp.), and willow [WI; *Salix schwerinii* E. L. Wolf (Salicaceae)]. In this study, we describe these groups of *A. malasiaca* adults as “host plant (MO, WI, and BB) populations”. Males are attracted to volatile chemicals from wounded plants of their respective host species that are released close to a female dummy^8^,^9^,^10^,^11^. In an MO population, sesquiterpene hydrocarbons, such as *β*-caryophyllene and *α*-humulene, may attract mate-seeking males^8^,^9^,^12^. In a WI population, nerol may have the same function^10^. In a BB population, *β*-caryophyllene, (*E*)-phytol, and sulfur attract males^11^. These chemicals are equally emitted from artificially wounded branches and branches damaged by the adults’ feeding^8^. Additionally, volatiles from their original host plants attract males but not females^8^,^9^,^10^,^11^. Therefore, we hypothesized that these volatiles may indicate the presence of conspecific females^9^,^10^,^12^. Because many of their hosts are sympatric, these beetles may encounter potential mates on other host plant species in addition to their original host. Understanding the differences in responses among populations could provide substantial insights into the background and mechanisms of natural host shifts.

When a male *A. malasiaca* encounters a female, a mating sequence occurs^13^. The male frequently touches the female’s elytra, which is covered with contact sex pheromones. Subsequently, the male holds and mounts the female, and then usually attempts to bend his abdominal tip toward her abdomen. Female elytra extracts also evoke male mating behaviour, and *A. malasiaca* contact sex pheromone components have already been identified in females of an MO population^14^,^15^,^16^,^17^.

We previously observed abdominal-bending behaviours of males from the three wild populations (MO, BB, and WI) toward glass dummies coated with elytra extracts from females of the three populations^18^. We confirmed a difference in male response sensitivity among the populations. MO population males were highly responsive to female extract-treated dummies, regardless of the female’s origin. However, WI and BB population males were less reactive to the MO population’s female extract. During experiments on the precopulatory responses of males toward female extract-treated dummies, we observed that considerable numbers of males in the BB and WI populations turned away from dummies coated with the extract of the MO population’s females immediately after touching their antennae to the dummies. Female elytra extracts from the three populations all contained contact sex pheromone components^18^. These prior findings indicated that females in the MO population probably possess extra chemicals that repel males from other feeding populations. These chemicals may induce the male rejection behaviours, regardless of the presence of contact sex pheromones, which in turn results in no mating attempt.

In this study, we focused on the rejection responses of male *A. malasiaca* from different host populations. We carried out analyses and identifications of chemicals that induced the male rejection response. To avoid complications from only using field-collected individuals, we also designed experiments with laboratory-reared individuals that were fed the same diet (a mulberry leaf-based artificial diet) during their larval stage and were then fed WI or MO after adult emergence. Because BB-fed individuals were weak and had relatively shorter lifespans (see a related manuscript file, Fujiwara-Tsujii *et al.* 2016), we used WI- and MO-fed individuals for the majority of the experiments. Based on the obtained results, we discuss the survival strategy and possibility of ongoing host changes in *A. malasiaca* in the field.

## Results

### Rejection responses of field-collected males to the extracts of females from three different host plant populations

The frequencies of the rejection responses of the MO population’s males to all of the female extracts were low (Table 1). However, the rejection response of the BB population’s males to the MO population’s female extract was significantly higher than to the BB and WI population’s female extracts (Table 1). This was also true for WI population males. The rejection responses to MO-female extracts were significantly higher than to the BB and WI population’s female extracts (Table 1).

### Laboratory-reared WI-fed male responses to extracts from WI- or MO-fed females

**Figure 1:**
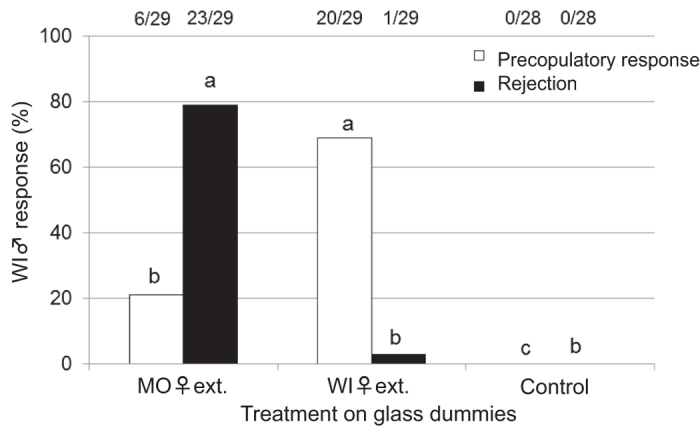
Precopulatory and rejection responses of WI-fed males to MO- or WI-fed female extracts. Open column, precopulatory responses; closed column, rejection responses. Response values accompanied by the same letter did not significantly differ at the *P* = 0.05 level (*n* × 2 chi-squared test and subsequent paired chi-squared test with Bonferroni-corrected *P-*values). The values at the top of the graph indicate the number of individuals that responded out of all of the replicates (responded/replicates).

The frequency of the abdominal bending response of WI-fed males to the WI-fed female extract was significantly higher than that to the MO-fed-female extract (Fig. 1). However, the frequency of the male rejection response to the MO-fed female extract was significantly higher than that to the WI-fed female extract and control.

### Rejection responses of WI-fed males to fractions of the MO-fed female extract

**Figure 2:**
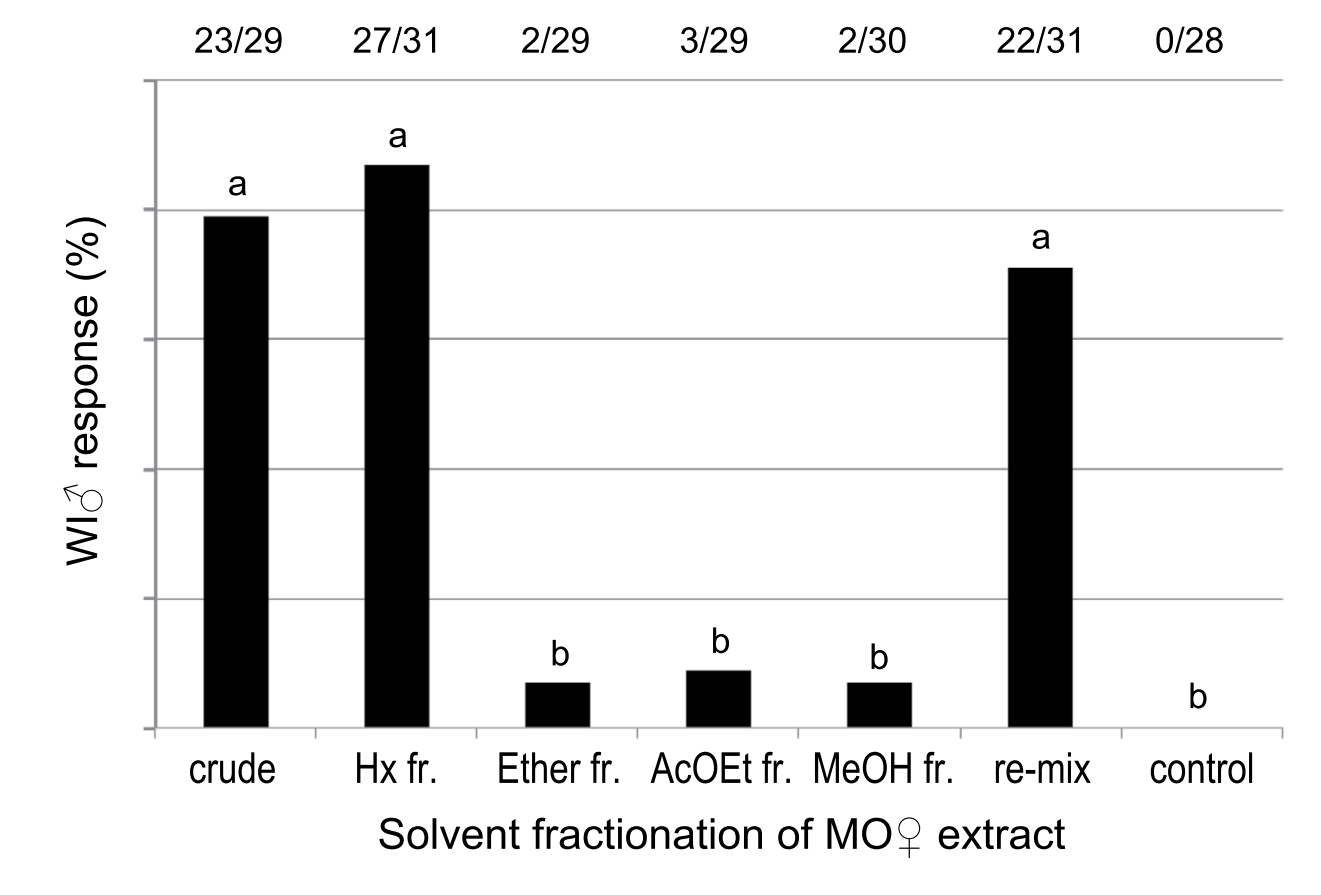
Rejection responses of WI-fed males to separated fractions of MO-fed female extracts. Hx fr.: *n*-hexane fraction; AcOEt: ethyl acetate, MeOH: methanol, re-mix: mixture of *n*-hexane, ether, ethyl acetate, and methanol fractions. Response values accompanied by the same letter did not significantly differ at the *P* = 0.05 level (*n* × 2 chi-squared test and subsequent paired chi-squared test with Bonferroni-corrected *P-*values). The values at the top of the graph indicate the number of individuals that responded out of all of the replicates (responded/replicates).

The frequency of the rejection response of WI-fed males to the MO-fed female extract’s *n*-hexane fraction was significantly higher than to other fractions. Additionally, it was similar to the responses to crude extracts and re-mixed fractions (Fig. 2).

### Gas chromatograph/mass spectrometry (GC/MS) analysis of the MO- and WI-fed female extract’s *n*-hexane fractions

**Figure 3:**
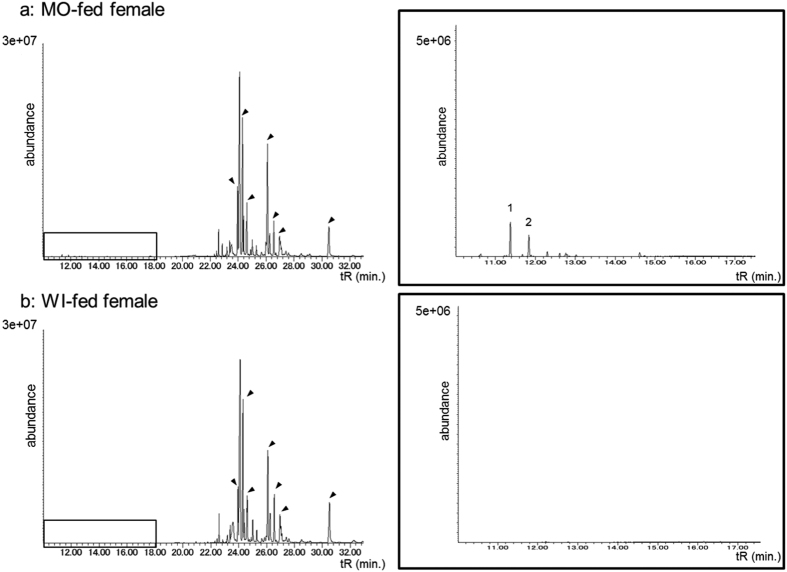
Total ion chromatogram of the *n*-hexane fractions separated from MO- (**a**) and WI-fed female (**b**) extracts. Enlarged chromatograms for each female are shown on the right. The peaks indicated with arrows were previously identified as contact sex pheromones^12^. Peaks 1 and 2 represent *β*-elemene and *β*-caryophyllene, respectively.

To identify characteristic chemicals only found in the MO-fed females, we analysed the MO- and WI-fed female extract’s *n*-hexane fractions using GC-MS. Comparisons of total ion chromatograms revealed two peaks in the extract from the MO-fed females that were absent in the extract from the WI-fed females (Fig. 3). Based on a database search (NIST), these peaks were the sesquiterpene hydrocarbons *β*-elemene (peak 1, major peak) and *β*-caryophyllene (peak 2, minor peak). The GC retention time and mass spectral data of the natural compound matched those of an authentic sample. Because elemene has enantiomers, we also analysed by GC-MS with a chiral column. The retention times and mass spectral data of natural elemene and authentic (−)-*β*-elemene were the same, however, we were unable to compare with another enantiomer, they cannot be sure that they were indeed the same enantiomer.

The MO-fed female extract contained 40 ng per individual of *β*-elemene (one female equivalent = 1 fe). The MO-twig extract also contained *β*-elemene, as reported in our previous study^9^. The large peaks that eluted later are the cuticular hydrocarbons, and the arrows in Fig. 3 indicate contact sex pheromone components as reported in our previous study^14^.

### *β*-Elemene’s effect on the WI-fed male rejection response

**Figure 4:**
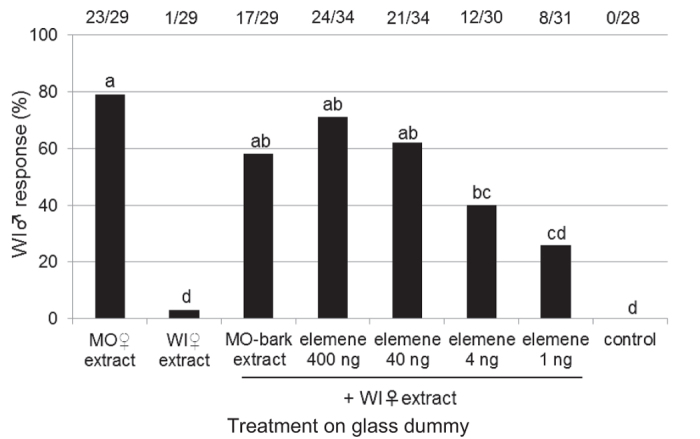
Effects of *β*-elemene and an MO-bark extract on WI-fed male rejection responses. Response values accompanied by the same letter did not significantly differ at the *P* = 0.05 level (*n* × 2 chi-squared test and subsequent paired chi-squared test with Bonferroni-corrected *P-*values). The values at the top of the graph indicate the number of individuals that responded out of all of the replicates (responded/replicates).

*β*-Elemene was blended with the WI-fed female extract on glass dummies and then exposed to WI-fed males. The rejection response frequencies of the WI-fed males to *β*-elemene-treated dummies decreased in a dose-dependent manner (40 ng = 1 fe; Fig. 4). The MO-bark extract, which contained *β*-elemene^9^, was also blended with the WI-fed female extract on a glass dummy. The associated rejection response of WI-fed males was similar to the response when exposed to the MO-fed female extract (Fig. 4). *β*-Caryophyllene, a minor specific component in the MO-fed female extract did not induce a rejection response from the WI-fed males (see Supplementary Fig. S1).

### The influence of host plant changes during adulthood on male rejection responses to the MO-fed females

**Figure 5:**
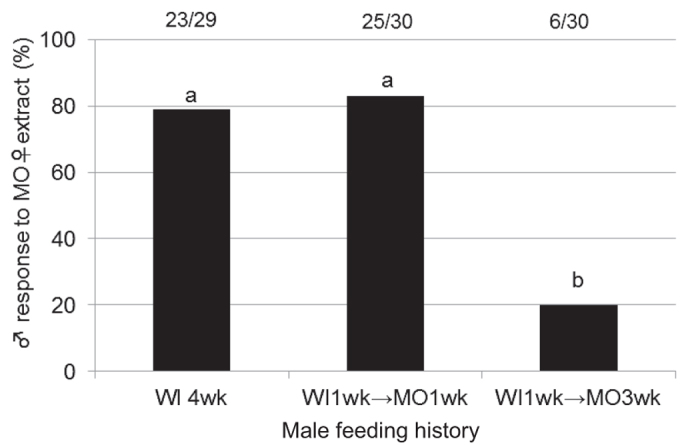
Rejection responses of males after host plant changes to the MO-fed female extract. wk; week. Response values accompanied by the same letter did not significantly differ at the *P* = 0.05 level (*n* × 2 chi-squared test and subsequent paired chi-squared test with Bonferroni-corrected *P-*values). The values at the top of the graph indicate the number of individuals that responded out of all of the replicates (responded/replicates).

To evaluate how host plant changes affect the male rejection response, we observed behavioural responses of males that were fed MO for 1 or 3 weeks after being fed WI for 1 week (WI1wk → MO1wk- or WI1wk → MO3wk-fed males). The rejection response frequency of WI1wk → MO1wk-fed males to the MO-fed female extract was similar to that of WI4wk-fed males (Fig. 5). However, the rejection response frequency of WI1wk → MO3wk-fed males was significantly lower than that of WI4wk-fed and WI1wk → MO1wk-fed males.

## Discussion

Asian longhorn beetles use various plant species as hosts; however, the relationships among the feeding populations have not yet been elucidated. In this study, we found that *A. malasiaca* males rejected female dummies treated with conspecific female extract that possessed *β*-elemene aquired from the female’s host plant. We observed this phenomenon between MO-fed females and different host plant-fed males. In most cases, *A. malasiaca* males initiate mating behaviour^13^, although some females of this species reject the males that attempt copulation. However, once the males rejected the females, the males never attempted to catch or copulate with those females, and the mating opportunity was lost. Our results represent the first example of a host-plant derived compound that appears to make females repellent to subsets of conspecific males that were not reared on the same host.

We observed and compared the male rejection behaviours toward dummies treated with female extracts from two host plants (MO and WI) in laboratory-reared *A. malasiaca* adults. The results were consistent with those of the field population analysis (Fig. 1 and Table 1). These results indicated that chemicals in the MO-fed female extract induced the rejection behaviour in the males. Because the MO-fed female extract’s *n*-hexane fraction from crude extract had the strongest activity (Fig. 2), we compared the *n*-hexane fractions from MO- and WI-fed female extracts using GC-MS (Fig. 3). Among the MO-fed female-specific chemicals, *β*-elemene was confirmed to be a major component. The addition of *β*-elemene or the MO-bark extract to the WI-fed female extract induced the rejection behaviour in WI-fed males (Fig. 4). Our research group previously identified *A. malasiaca* contact sex pheromone components^12^. Males can recognize sexually mature females based on the contact sex pheromones on her elytra^18^. However, the *β*-elemene in her elytra seems to strongly induce the rejection behaviour in males, even if she has an otherwise attractive blend of contact sex pheromone components. In this study, we added 10, 1, 0.1, or 0.025 fe of *β*-elemene to the WI-fed female extract, and the rejection activity was not significantly different among the two higher concentrations and was similar to that of the MO-fed female extract (Fig. 5). Thus, 1 fe of *β*-elemene was sufficient to cause rejection in WI-fed males.

In the field, the MO population’s males rejected the dummies treated with the MO population’s female extract much less frequently than did the WI and BB populations’ males (Table 1). Additionally, an analysis of the laboratory-reared adults revealed that the MO-fed males showed a relatively low frequency of rejection behaviour toward the MO-fed female extract (Fig. 1), which indicates that *β*-elemene does not cause the rejection response in MO*-*fed males. In the field, the WI and BB populations’ females were attractive to the males of all of the host plants; however, the MO population’s females were only attractive to males from the same population. These results indicate that the continuous feeding on MO as a host plant affects mating choices.

To determine if a host plant change affects the male rejection response, we conducted assays using males with different feeding experiences. We took three groups of adult males fed MO for 1 or 3 weeks after being fed WI for 1 week (WI1wk → MO1wk or WI1wk → MO3wk), and those fed WI for 4 weeks (WI4wk), and observed their reactions to the MO-fed female’s extract. A high frequency of rejection responses was maintained in the WI1wk → MO1wk-fed males when exposed to the MO-fed female extract (83%); however, this was significantly lower if they were fed MO for 3 weeks (males of WI1wk → MO3wk-fed, 20%; Fig. 5). This result indicates that host plant acclimation occurred gradually after changing host plant.

After switching host plants, males may take a relatively long time to acclimatise to a new host plant, but once they do, it is possible to find and copulate with mates on this new host plant. Their adult lifetime is approximately 3 months in the field, and that of three-weeks occupies one-fourth of its mating season. Currently, we do not know why this maladaptive situation is occurring, however, it brings considerably negative effect on their reproductive strategy. We speculate that a host change in this species does not occur very often, it occurs only when the original hostplant condition becomes bad or not adequate for reproduction. For example, Fujiwara-Tsujii *et al.* (see a related manuscript file) reported that BB is not a suitable host plant for reproduction, although *A. malasiaca* adults were collected in BB gardens; therefore, members of BB populations may change host plants during the adult stage.

In field populations, males were attracted by odours of the plant species on which it originated^8^,^9^,^10^,^11^. In laboratory experiments, males were attracted to the plant species on which they had most recently fed or to plants with which they had previous experience^19^, indicating that a male would be more likely to approach females that fed on his original host plant than females that fed on other host plants. In this study, we found that *β*-elemene acted to reject WI-fed males that touched MO-fed females. In addition, WI-fed males required 3 weeks of continuous feeding on MO to lose their aversion to MO-fed females (Fig. 5). Therefore, prior host plant experience largely affected both male orientation and mate recognition.

The preference of females for MO is also noteworthy. Female *A. malasiaca* chose and mostly consumed MO if given the three host plants simultaneously^19^, and MO had the greatest effect, of the three host plant species, on their reproductive properties (see a related manuscript file). The strong preference of females for MO and the male preference for their most recent host plants could lead to mate incompatibility. If this phenomenon confirmed in semi-field or field conditions, there is the possibility to apply a simple vegetation management-like control for this beetle. For example, the introduction of citrus trees into other host plants’ orchards could lead to fewer mating opportunities. Females of *A. malasiaca* change their host plant easily to MO, whereas the males hardly to change their hostplant to MO^19^. Consequently, the impact of the male rejection behaviour towards the MO females on the reproductive output in such scenarios should be investigated.

A male-produced attractant pheromone was identified in *A. glabripennis*^20^,^21^ and recently in *A. chinensis*^22^. New flight traps were recently developed to efficiently capture these cerambycid beetles^23^. Any attractant pheromone was not found in *A. malasiaca* yet, so we will confirm whether they have a same kind of male-produce pheromone or not in the next season. Combinations of these attractant pheromone and host plant derived attractant chemicals with efficient flight traps will lead strong monitoring methods. As for *β*-elemene, it will be difficult to use for monitoring, however, useful for protect young, nursery trees (except for mandarin orange) from feeding damage by this species when this chemical is sprayed on the plants.

From our previous works, the male’s preference for the most recently fed-on host plant is a “learned type of response”. Their responses to host plants were not affected by what they were reared on before their eclosion^19^. However, females from the field MO population strongly preferred their original host. Reared adult females (offspring of the MO population) also preferred MO regardless of their most recent host^19^. It is unknown whether the high preference for MO is inherited or not, and why it apparently occurs only in females. To investigate these issues, we will attempt to rear offspring of the WI population.

## Methods

### Adult insect collection and rearing

Adult *A. malasiaca* were collected by hand from their host plants. The MO population was sampled from MO groves on Kunisaki Peninsula, Oita Prefecture, Japan in mid-June of 2011 and 2014. The WI population was sampled from WI mesh-net houses in Date, Fukushima Prefecture, Japan in early July 2011. The BB population was sampled from BB (*Vaccinium* spp.) gardens in Shimoina, Nagano Prefecture, Japan in July 2011. All of the populations were collected at the beginning of the adult emergence season. Beetles were individually reared in clear plastic cups (~11 cm diam. × 9.5 cm height) at 25 °C under a 15L:9D photoperiod illuminated by fluorescent lamps. Each beetle was fed branches of its original host plant until it was used in bioassays or freeze-killed for extraction.

### Plants for feeding

MO and BB branches were field collected in the regions where the beetles were sampled and then transported to the National Institute of Agrobiological Sciences (NIAS) laboratory. WI branches were collected from trees that were grown at the NIAS. All of the cut branches were stored at 5 °C and used within 10 d.

### Egg collection and laboratory rearing to adults

*A. malasiaca* eggs were obtained from the females of MO population. Approximately 200 female adults were collected from MO groves in Kunisaki Peninsula, Oita Prefecture, Japan in mid-June of 2014. Because considerable amounts of eggs were needed to rear to adults in the laboratory, MO population was the only population we could obtain many females. Eggs laid in the MO bark were collected and rearing of larvae to adult was performed as described in Fujiwara-Tsujii *et al.* (see a related manuscript file). All larvae were reared on the same artificial diet (Silkmate 2S, mulberry leaf-based diet, Nihon Nosan Kogyo, Japan) throughout their larval stages. Emerged adults were individually contained in transparent plastic cups (~11 cm diam. × 9.5 cm height). Adults start feeding on host branches 1 week after adult emergence^19^; therefore, 1-week-old adults were divided into two groups, one of which was given MO branches and the other given WI branches.

### Female elytra extract from field-collected and laboratory-reared adults

Thirty days after collection in the field, females of the three populations were killed by freezing at −30 °C. For the laboratory-reared adults, females were killed after feeding on host plant branches for 3 or 4 weeks by freezing at −30 °C. The elytra of 10 females from each population and group were separately collected and were placed in 15 mL of ether (1.5 mL/female, distilled just before use). After 5 min at room temperature, the extract was decanted. Elytra were rinsed twice with 15 mL of ether, and the rinses were added to the extract. Ether was removed from the extracts under reduced pressure at below 30 °C, and the resulting residue (the “female extract”) was dissolved in 200 μL of *n*-hexane and stored at −30 °C until use.

### MO bark extraction

Twenty-five grams of MO bark taken from freshly cut branches was placed in 100 mL of ether (0.25 g/mL). After 5 min at room temperature, the extract was decanted. The bark was rinsed twice with 100 mL of ether, and the rinses were added to the extract. Ether was removed from the extracts under reduced pressure at below 30 °C, and the resulting residue (the “MO-bark extract”) was dissolved in 100 mL of *n*-hexane and stored at −30 °C until use.

### Male responses to the female extracts of three field populations from different host plants

Behavioural assays were conducted from the 8^th^ to 16^th^ of August 2011. Behavioural activity was evaluated as described in Fukaya *et al.*^13^ and took place between 13:00 and 16:00 JST (9–12 h after lights on). Capsule-shaped black glass rods (12-mm diam. × 35-mm length) were used as female dummies. Each dummy was affixed with a small piece of adhesive tape to the centre of a filter-paper disc (15-cm diam., Toyo No. 2, Toyo Roshi Kaisha, Tokyo, Japan) and coated with female extract (1 fe) dissolved in approximately 20 μL of *n*-hexane.

We tested groups of males from each population with each female extract. In each test, a male beetle was placed nearby and allowed to contact the dummy with his antennae. Behavioural responses were observed for 5 min after the first contact. Male responses were categorized as follows: abdomen bent down toward the dummy surface (=abdominal bending)^18^ or moved away from the dummy after touching it with his antennae (=rejection). If a male did not react to the dummy, he was gently guided until his antennae contacted the dummy at least three times during the observation period. Sample sizes for this assay were as follows: MO and WI males, 30; WI males vs. MO-female extract, 22; and WI males vs. BB- and WI-female extracts, 21.

### Precopulatory and rejection responses of WI-fed males to MO- or WI-fed female extracts

Behavioural assays were conducted from June to July 2015. Behavioural activity was evaluated as described above. We tested WI-fed males with dummies treated with MO- or WI-fed female elytra extracts, or MO-fed female extract fractions (*n*-hexane, ether, ethyl acetate, and methanol fractions from silica-gel column chromatography, and a re-mixed solution containing all of the fractions). Male responses were categorized as precopulatory response or rejection. Males were also presented with an untreated dummy as a control. The sample sizes of the assays are shown in Figs 1 and 2.

### Column chromatography

The column chromatography of MO- and WI-fed female elytra extracts from laboratory-reared individuals was carried out on silica gel columns (particle size: 75–150 μm, Wako Gel C-200, Wako Pure Chemicals, Osaka, Japan). The solvents used were analytical grade. Each female elytra extract (10 fe) was individually applied onto silica gel (0.8 g SiO_2_ each), and compounds were successively eluted with 16 mL of *n*-hexane, ether, ethyl acetate, and methanol. Each fraction was concentrated to ~200 μL and stored at −30 °C until use.

Column chromatography of the MO-bark extract was also performed based on the above-described procedure. The MO extract was applied onto the silica gel and eluted with *n*-hexane.

### GC/MS analyses

GC/MS analyses were performed on an HP Agilent 6890N GC (Agilent, Santa Clara, CA, USA) equipped with a split/split-less injector and an apolar capillary column DB-5MS (30 m × 0.25 mm ID × 0.25 μm film thickness; Agilent) linked to a 5973 mass selective detector. Helium was the carrier gas at a constant flow rate of 1.1 mL/min. The injection port was kept at 200 °C, and the ion source temperature was 220 °C. The GC oven temperature was held at 60 °C for 1 min, then increased from 60 °C to 280 °C at 10 °C/min and was finally held at 280 °C for 10 min.

*β*-elemene was found in the *n*-hexane fraction and is the candidate repellent from MO females. Elemenes can form from structural rearrangements under GC conditions (Cope rearrangement of germacrenes to elemenes)^24^,^25^. To prevent false *β*-elemene identification, on-column injections were also used for analysis. On-column injections were made into the GC column at 53 °C. The GC oven temperature was held at 50 °C for 1 min, increased from 50 °C to 230 °C at 10 °C/min, and held at 230 °C for 10 min.

### Effects of *β*-elemene and an MO-bark extract on WI-fed male rejection responses

The candidate chemical, (−)-*β*-elemene (LKT Laboratories, Inc., St Paul, MN, USA; purity, 98.9%), was adjusted to 0.025, 0.1, 1, and 10 fe (1, 4, 40, and 400 ng per treatment, respectively) and applied to dummies along with the WI-fed female extract. As described in Yasui *et al.*^9^, *β*-elemene was identified from the MO-bark extract. To confirm that MO bark is the source of *β*-elemene, the MO-bark extract was adjusted to possess the same amount of sesquiterpene hydrocarbons for a single fe (3 μg)^9^ and applied to dummies along with the WI-fed female extract. WI-fed male rejection responses to the treated dummies were observed. The sample sizes of the assays are shown in Fig. 4. We also analyzed *n*-hexane fractions of the MO-fed female extract and MO-bark extract, and purchased *β*-elemene with a chiral column (RT-b-DEXm, 30m × 0.25 mm ID × 0.25 μm film thickness; Restec, Bellefonte, PA, USA), and the retention times and the mass spectra of those were identical.

### Rejection responses of males after host plant changes to the MO-fed female extract

To evaluate how host plant changes affect the male rejection response, after 1 week of WI feeding, males were fed MO for 1 or 3 weeks (WI1wk → MO1wk-fed or WI1wk → MO3wk-fed, respectively). They were then exposed to dummies treated with the MO-fed female extract. Four-week-fed WI males were also included for comparison. The sample sizes of the assays are shown in Fig. 5.

### Statistical analyses

Behavioural assay data were first analysed with an *n* × 2 chi-square test. If this test was significant, then a paired chi-square test was subsequently calculated with Bonferroni-corrected *P-*values^26^. In the figures, values accompanied by the same letter did not significantly differ at the *P* = 0.05 level.

## Additional Information

**How to cite this article**: Yasui, H. and Fujiwara-Tsujii, N. Host plant affects the sexual attractiveness of the female white-spotted longicorn beetle, *Anoplophora malasiaca. Sci. Rep.* **6**, 29526; doi: 10.1038/srep29526 (2016).

## Supplementary Material

Supplementary Information

## Figures and Tables

**Table 1:** Frequencies of rejection responses (%) of male *A. malasiaca* from MO, BB, and WI field populations to glass dummies treated with elytra extracts from field-collected females of the same population.

**Populations**	**Rejection responses**		
	**MO ♀**	**BB ♀**	**WI ♀**
MO ♂ ^1^	7 ^a^_B_	0 ^a^_A_	0 ^a^_A_
BB ♂ ^2^	70 ^a^_A_	0 ^b^_A_	3 ^b^_A_
WI ♂	50 ^a^_A_^3^	10 ^b^_A_^4^	5 ^b^_A_^4^

MO ♂, N = 30; ^2^ BB ♂, N = 30; ^3^ WI ♂, N = 22; ^4^ WI ♂, N = 21. Values with the same lower-case superscript letter in a row (male behaviour) or the same upper-case subscript letter in a column (female extracts) did not significantly differ at the *P* = 0.05 level based on an *n* × 2 chi-squared test or the subsequent paired chi-squared test with Bonferroni-corrected *P-*value.
