# Supplementary figures and images for: Host plant affects the sexual attractiveness of the female white-spotted longicorn beetle, Anoplophora malasiaca
Source: Sci Rep. 2016 Jul 14;6:29526. doi: 10.1038/srep29526 (PMC4944169; doi:10.1038/srep29526)

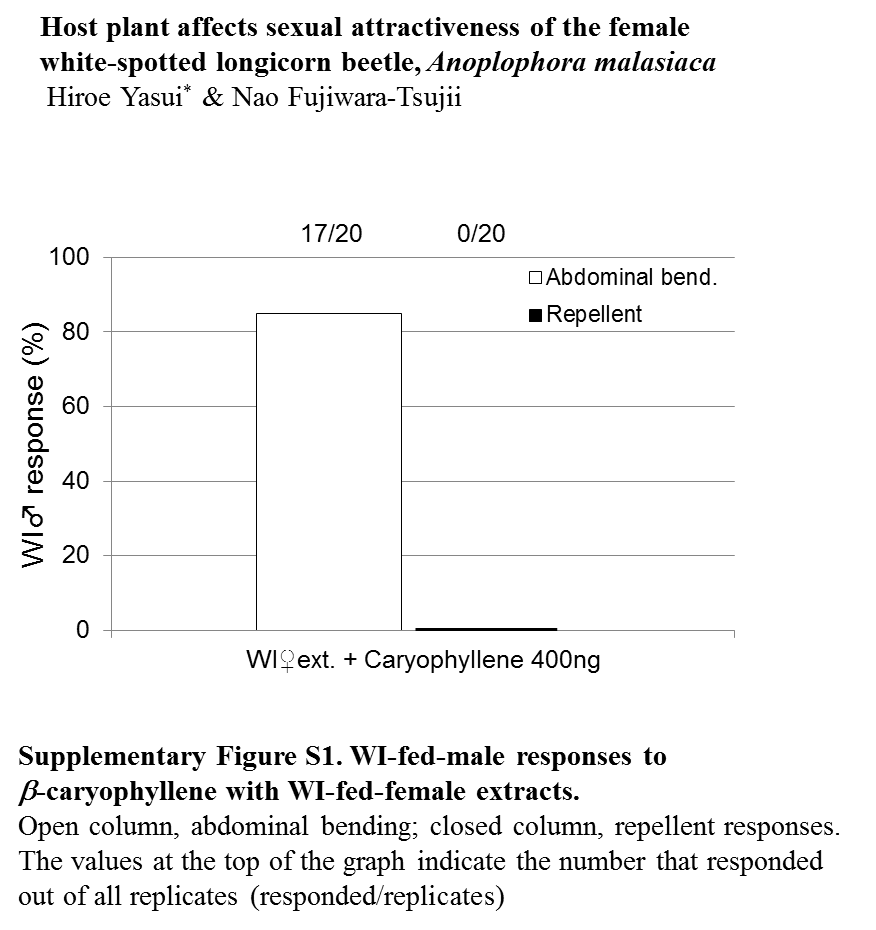

Supplement: Supplementary Information [file srep29526-s1.doc]
